# Learning Causal Effects From Observational Data in Healthcare: A Review and Summary

**DOI:** 10.3389/fmed.2022.864882

**Published:** 2022-07-07

**Authors:** Jingpu Shi, Beau Norgeot

**Affiliations:** Anthem, Inc., Point of Care AI, Palo Alto, CA, United States

**Keywords:** electronic health record, causal inference, machine learning, healthcare, treatment effects, review, potential outcome framework, patient population

## Abstract

Causal inference is a broad field that seeks to build and apply models that learn the effect of interventions on outcomes using many data types. While the field has existed for decades, its potential to impact healthcare outcomes has increased dramatically recently due to both advancements in machine learning and the unprecedented amounts of observational data resulting from electronic capture of patient claims data by medical insurance companies and widespread adoption of electronic health records (EHR) worldwide. However, there are many different schools of learning causality coming from different fields of statistics, some of them strongly conflicting. While the recent advances in machine learning greatly enhanced causal inference from a modeling perspective, it further exacerbated the fractured state in this field. This fractured state has limited research at the intersection of causal inference, modern machine learning, and EHRs that could potentially transform healthcare. In this paper we unify the classical causal inference approaches with new machine learning developments into a straightforward framework based on whether the researcher is most interested in finding the best intervention for an individual, a group of similar people, or an entire population. Through this lens, we then provide a timely review of the applications of causal inference in healthcare from the literature. As expected, we found that applications of causal inference in medicine were mostly limited to just a few technique types and lag behind other domains. In light of this gap, we offer a helpful schematic to guide data scientists and healthcare stakeholders in selecting appropriate causal methods and reviewing the findings generated by them.

## Introduction

In healthcare, it is important to distinguish between association and causation when we study treatment effects on patient outcomes. Association between two variables is non-directional and implies that the two variables are correlated. In contrast, causation is directional and indicates that one variable causes the other. In clinical studies, we are more interested in causal analysis to reveal whether a treatment causes a desired outcome.

Using observational data to infer causal treatment effects has become popular in the past decade due to two pivotal advances: the increasingly available patient data captured in Electronic Health Records (EHRs) and machine learning techniques that can efficiently and intelligently analyze large-scale data. On the data side, health care providers worldwide have widely adopted EHRs (1, 2), which capture patients' clinical and demographic information during interactions with health systems. In addition to EHRs, patient claims data are increasingly available to improve models in the healthcare domain (3). On the algorithm side, machine learning models such as artificial neural networks are powering online search engines, shopping websites, and recommender systems (4). These machine learning models are increasingly used to improve causal inference algorithms.

In the past, many different schools of learning causality coming from different fields of statistics resulted a fractured state of causal inference, creating confusion about which algorithm to use in a study. Recently, the intersection of causal inference, machine learning, and patient data has formed a new front in clinical research. Accordingly, many traditional causal inference models have been improved and many new models have been proposed. While this has enhanced the number of model options to select from in causal inference studies, it has also led to even greater confusion about which type of algorithm is appropriate for a given application. Lack of systematic knowledge of which approaches are promising in theory vs. the approaches that have been validated through real world applications further complicates the debate.

There are different stakeholders in healthcare, including healthcare providers, administrators, clinical researchers, data scientists, and many others. While data scientists, computer engineers, and biomedical statisticians may be less prone to such confusion, the fractured state in this field makes it difficult for other participants to understand the many different types of models and intuitively interpret the model results. We believe it is imperative to address this confusion for all healthcare participants to unlock the massive potential to improve patient outcomes that could be obtained by studying the causal effects of interventions from large-scale, representative, observational patient data that is now available.

In this review, we start by explaining the broad and heterogenous fields of causal inference. We then distill all of these techniques down into a simple unified framework of three algorithm families, based on size of the target patient population that the causal effect estimation will be applied to. This simple unified frame based on the size of the target patient population is important: while statisticians in medical informatics may not necessarily group the algorithms this way, it is beneficial for frontline healthcare professionals such as doctors and nurses to understand the drug effect in the context of its target population, and the effect's variance and bias characteristics when the drug is applied to the treated patient. From the perspective of this unified framework, we then review all existing applications of causal inference in healthcare in the literature, and identify key components of causal inference that are, as of now, lacking in the healthcare domain. Finally, we use these insights to create an intuitive schematic to guide researchers and stakeholders through the process of selecting an appropriate causal inference technique based on their study objectives.

This review is an extension of several works in previous literature on observational causal inference. For example, the authors in Yao et al. (5), Guo et al. (6), and Ding and Li (7) reviewed causal inference in general but without a focus on clinical settings. The authors in Landsittel et al. (8) offered a narrative review of basic concepts of causal inference but did not consider new developments in this field. Prior reviews (9–11) have narrowly focused on the matching method of causal inference, while in this paper we expand to include a much broader algorithm types.

We conclude this section by providing below a summary of all the approaches we review, with respect to their variance-bias trade-off, advantages, disadvantages, and how widely they are applied in clinical studies.

## Causal Inference Assumptions, Frameworks, and Target-Population Intervention Sizes

### Confounding Variables

Causal inference differs from associative studies due to the modeling of confounding variables (covariates), defined as variables that affect both the treatment and the outcome. In associative studies which focus on patient outcome estimates, confounding variables are modeled in an inclusive manner because the inclusion of these variables in the model improves estimate accuracy. In contrast, causal inference which reveals the causal relationship between treatments and patient outcomes models the confounding variables in an exclusive manner in that their effects are removed through various approaches we review in this paper.

### Assumptions

In the literature, several assumptions are widely adopted in causal inference (12). The unconfoundedness assumption, also known as ignorability, states that all confounding variables are observed in the data. In practice, domain experts often examine as many patient variables as possible, including their demographic and clinical characteristics, so that this assumption can be met. The common support or positivity assumption states that any patient has a non-zero probability of being present in any of the treatment groups. The validity of this assumption can be checked by calculating the patients' propensity scores (12). The Stable Unit Treatment Value assumption (SUTVA) states that a patient's outcome only depends on the treatment this patient receives, and not affected by the outcome or treatment of any other patients. The consistency assumption links the potential outcomes to the observed data and implies that the potential outcome under an observed exposure is precisely the outcome that is observed (13).

### Bias-Variance Tradeoffs Based on Target-Population Intervention Sizes

Researchers, clinicians, and other healthcare stakeholders may wish to know the treatment effects at different population levels for different purposes. For example, they may want to evaluate the overall effectiveness of the treatment on the whole population. They may want to understand treatment effect differences in different subpopulations to identify the subpopulation where the treatment is the most effective or least effective. When they treat an individual patient, they may want to know the individual-level treatment effects considering the patient's unique medical benefits and risks.

Driven by such needs, researchers conduct causal inference at different target-population intervention sizes: at one end of the spectrum is the Average Treatment Effect (ATE) that captures the treatment effect for a population at large; at the other end is the Individual Treatment Effect (ITE) that captures the treatment effect heterogeneity across individuals; in between is the conditional average treatment effect (CATE) that captures the treatment effect for subpopulations.

In clinical practices, at the receiving end of any treatment are individual patients. Correspondingly, different treatment effects (ATE, CATE, and ITE) are eventually applied to individual patients. Therefore, it is important to understand the variance-bias tradeoff of the estimate at different target-population intervention sizes: if we use ATE as the treatment effect for an individual patient, the bias will be high due to effect heterogeneity across patients in the population, but the variance will be low due to more data being used in the inference; in contrast, if we use ITE for a patient, the bias will be low, but the variance will be high.

As the rest of the paper shows, ATE provides the best option and fosters estimate efficiency for the whole population, but may not provide the most accurate estimate for any individual patient. ITE maximally leverages the data, but risks being uninterpretable to clinical practitioners. CATE represents a balance between bias and variance and tracks the clinical definition of patient subgroups.

### Two Frameworks

There are two widely accepted frameworks in the literature for causal inference: the structural causal model (SCM) (14–16) and the potential outcome framework (POF) (12, 17, 18). SCM consists of two components, the causal graph and the structural equations. A causal graph is a directed acyclic graph (DAG) where the edges represent causal relationships, and the nodes represent variables including treatments, outcomes, and covariates that may or may not be observed. Causal effects can be quantitatively specified through a set of structural equations.

The DAG and structural equations together provide a comprehensive theory of causality and seamlessly tie essential concepts and methodologies in causal inference (14, 19, 20). In addition, it can possibly deal with cases where confounders cannot be measured. For example, in Barter (21), the author used the blood type as an instrument variable—defined as a variable that affects the outcome only through the treatment variable—to estimate the average survival benefit from receiving a liver transplant.

The other framework, called the potential outcome framework, centers on the concept of potential outcomes. In the simplest term, potential outcomes are all the possible outcomes for a patient under all possible treatments, with each outcome corresponding to a treatment. Note that only one potential outcome can be observed for a given patient at a given time. We call the potential outcome that would have been observed had the treatment been different the counterfactual or the missing outcome. In the simplest case, there is only one treatment to consider. A patient can be either given the treatment, i.e., assigned to the treated group, or given no treatment, i.e., assigned to the control group. Under the potential outcome framework, the treatment effect is the difference between the potential outcome if the patient is treated and that if the patient is not treated.

CSM and POF are not competing frameworks but can be unified (22). Despite this fact, the two frameworks have differences in what causal questions they are best suited to handle. Given its strong theoretical grounding, CSM is ideally suited to identifying unknown causal and confounding variables, as well as facilitating explanation. While it is useful to identify all the variables in the causal graph and their causal connections, the primary objective in healthcare is often to estimate the actual effect of a given treatment. POF is best suited for generating these estimates, because comparing potential outcomes eases the removal of confounding effects and enables a natural connection to traditional statistical analyses. For this reason, POF is more widely adopted for healthcare research and will be the focus of this review.

## Causal Inference Methods by Target-Population Intervention Sizes

In this section we review causal inference approaches in the literature under the potential outcome framework and the assumptions stated in Section Causal Inference Assumptions, Frameworks, and Target-Population Intervention Sizes. We organize our review by the approaches' target-population intervention size: from ATE for the whole population to CATE for subpopulations and ITE for individual patients.

We first explain some key notations. Suppose we are interested in the causal effect of a treatment *A* on outcome *Y*. The potential outcome denoted by *Y*^*a*^ is the outcome that we would observe under a possible treatment *A* = *a*. In a binary treatment case, *a* can possibly take on two values *a*∈{0, 1}, where 0 indicates the patient is not treated and 1 indicates the patient is treated. We denote the confounding variables by *X*. For simplicity, we only focus on the binary treatment case in this paper.

### Estimate ATE for the Whole Population

In the binary treatment case, the ATE estimate for the population can be calculated as


(1) $$\begin{array}{r} \tau =E\left(Y^{1}-Y^{0}\right)=E\left(Y^{1}\right)-E\left(Y^{0}\right) \end{array}$$

It is the difference between the expected potential outcomes of the population if everyone is treated (*A* = 1) and if no one is treated (*A* = 0).

Note that ATE cannot be directly calculated from equation (1) because only one of the potential outcomes, either $Y_{i}^{1}$or $Y_{i}^{0}$, can be directly observed for patient *i*, nor can it be directly calculated from the expected outcomes of the treated and control groups,


(2) $$\begin{array}{r} E\left(Y^{1}-Y^{0}\right)\neq E\left(Y|A=1\right)-E\left(Y|A=0\right) \end{array}$$

due to the existence of confounding variables *X*. In general, the distribution of confounding variables is different in the treated and control group. If their expected outcomes are directly compared to calculate treatment effects without adjusting for confounding variables, the calculated treatment effects would be biased.

#### Propensity Score-Based Approaches

Propensity score of a patient is the conditional probability that this patient with *X* = *x* is assigned to the treated group. It is expressed as


$$\begin{array}{l} \pi \left(x\right)\;=\;P_{r}\left(A=1\;|X=x\right), \end{array}$$

and can be estimated using models such as logistic regression (12). We can use the propensity score in three different ways to balance the covariate distribution between the treated and control group and thus make the two groups comparable.

The first way is to create new control and treated groups using propensity score matching (12, 23). The most straightforward approach is greedy one-to-one matching: one patient from the control group is matched to one patient from the treated group based on their propensity scores. Data of unmatched patients gets thrown away. The covariate distribution of the matched control and treated group is balanced. Then we can calculate the difference of the expected outcomes of the two new groups as the average treatment effect (ATE). In contrast to equation (2), the equation below is now correct due to balanced covariate distributions,


$$\begin{array}{l} {E\left(Y^{1}-Y^{0}\right)}_{balanced}={E\left(Y|A=1\right)}_{balanced}-{E\left(Y|A=0\right)}_{balanced} \end{array}$$

In addition to one-to-one matching, propensity score is used in other similar algorithms to create matched groups. These algorithms differ from each other in whether patients are chosen with or without replacement (24), whether matching is optimal, greedy (24), one-to-one, or one-to-many (25), and what metric is used to measure similarity between two patients (11, 23, 26, 27).

The second way of using propensity scores, known as Inverse Probability of Treatment Weighting (IPTW) (28), is to assign different patients with different weights in the calculation of ATE. For patient *i*, the weight is calculated as


$$\begin{array}{l} w_{i}\;=\;\frac{A_{i}}{P\left(A_{i}\;=\;1|X_{i}\right)}+\frac{1-A_{i}}{1-P\left(A_{i}=\;1|X_{i}\right)}. \end{array}$$

From this equation, we can see that if patient *i* is in the treated group (*A*_*i*_ = 1), the weight assigned to this patient is $w_{i}=\frac{1}{P\left(A_{i}=1|X_{i}\right)}=\frac{1}{\pi \left(x_{i}\right)}$. If the patient *i* is in the control group (*A*_*i*_ = 0), the weight then becomes $w_{i}=\frac{1}{1-P\left(A_{i}=1|X_{i}\right)}=\frac{1}{1-\pi \left(x_{i}\right)}$. The weight of a patient in a group is just the inverse probability of this patient being assigned to this group. The ATE of the population can then be calculated as


$$\begin{array}{l} \hat{\tau }\;=\;\frac{1}{n_{1}}\sum_{i}w_{i}y_{i}^{1}-\frac{1}{n_{0}}\sum_{i}w_{i}y_{i}^{0} \end{array}$$

where $y_{i}^{1}$ ($y_{i}^{0}$) is the observed outcome for patient *i* if this patient is treated (untreated), *n*_1_ and *n*_0_ are the number of patients in the treated and control group, respectively. Intuitively, the IPTW approach balances covariate distributions between the two groups by giving the patients underrepresented (overrepresented) in a group higher weight (lower weight).

The third way of using propensity score in ATE estimate is to stratify the population into subpopulations based on the propensity scores of the patients (29). The treatment effect from each subpopulation is then calculated and combined to estimate the ATE of the whole population.

Propensity score-based approaches are intuitive, easy to understand, and capable of producing an unbiased ATE estimates if the propensity score is correctly estimated. If the propensity models are misspecified (for example, the function form in the logistic regression is wrong), the propensity score estimates and subsequent ATE estimates would be biased.

#### Outcome Regression-Based Approaches

One fundamental challenge in causal inference is the missing data problem: only one of the potential outcomes is observable for a given treatment and patient. Regression models can be used to estimate the missing outcomes, thus solve the missing data problem (17, 30).

Here we outline how outcome regression models are used in ATE estimates but leave the detailed review of these models to Section Estimate ITE for Individual Patients. Suppose the outcome regression function for the control and treated group is *m*_0_(*X*) and *m*_1_(*X*), respectively. Once the two functions are fitted, the missing potential outcomes can be predicted as $\hat{Y^{0}}=m_{0}\left(X\right)$ and $\hat{Y^{1}}=m_{1}\left(X\right)$. The average treatment effect for the population can be estimated as,


(3) $$\begin{array}{r} \hat{\tau }=E\left(Y^{1}-Y^{0}\right)=\frac{1}{n_{0}+n_{1}}\overset{n_{0}+n_{1}-1}{\underset{k=0}{\sum }}\left(\hat{Y_{k}^{1}}-\hat{Y_{k}^{0}}\right) \end{array}$$

which first calculates the difference between the two predicted outcomes of each patient, then averages these differences over all the patients in both groups. Note that *m*_0_(*X*) and *m*_1_(*X*) can either take on the same function form, in which case the treatment assignment variable *A* must be explicitly included in the model as one of the independent variables, or take on different function forms, in which case *A* is excluded in the model.

Outcome regression models do not require an estimate of propensity scores. However, misspecification of the regression model (for example, the regression function form is wrong) can lead to biased treatment effect estimates.

#### Doubly Robust Estimator

Both the outcome regression and the propensity model can be misspecified. A combination of the two models, known as a Doubly Robust Estimator (DRE), is proposed in Robins et al. (31) and Funk et al. (32). It calculates the expected outcome for the treated and control group as


(4) $$\begin{array}{r} E\left(Y^{1}\right)=\frac{1}{n_{0}+n_{1}}\overset{n_{0}+n_{1}-1}{\underset{i=0}{\sum }}\left\{\frac{A_{i}Y_{i}}{\pi_{i}\left(X_{i}\right)}-\frac{A_{i}-\pi_{i}\left(X_{i}\right)}{\pi_{i}\left(X_{i}\right)}\;m_{1}\left(X_{i}\right)\right\} \end{array}$$

and


(5) $$E\left(Y^{0}\right)=\frac{1}{n_{0}+n_{1}}\sum_{i=0}^{n_{0}+n_{1}-1}\left\{\frac{(1-A_{i})Y_{i}}{1-\pi_{i}\left(X_{i}\right)}-\frac{A_{i}-\pi_{i}\left(X_{i}\right)}{1-\pi_{i}\left(X_{i}\right)}\;m_{0}\left(X_{i}\right)\right\}$$

respectively. Then the ATE can be estimated as *E*(*Y*^1^)−*E*(*Y*^0^). Essentially, this DRE is an IPTW estimator augmented by term $\frac{A_{i}-\pi_{i}\left(X_{i}\right)}{\pi_{i}\left(X_{i}\right)}\;m_{1}\left(X_{i}\right)$ in Equation (4) and term $\frac{A_{i}-\pi_{i}\left(X_{i}\right)}{{1-\pi }_{i}\left(X_{i}\right)}\;m_{0}\left(X_{i}\right)$ in equation (5). For this reason, it is also called an augmented IPTW estimator.

Another type of DRE is the Targeted Maximum Likelihood Estimator (TMLE), initially proposed in Laan and Rubin (33) and further studied in Schuler and Rose (34). In this approach, an outcome regression model is first used to estimate *E*(*Y*|*A, X*), which is then updated using estimated propensity score π(*X*) in the so called “targeting” step, yielding a better estimate E^*^(*Y*|*A, X*). Average treatment effect can be calculated as E^*^(*Y*^1^)−E^*^(*Y*^0^).

As implied in the name, DREs have a nice doubly robust property that ensures the ATE estimate is unbiased if only the outcome regression model or only the propensity model is correct. These models also tend to be more efficient than just the IPTW estimators.

### Estimate CATE for Subpopulations

In some cases, researchers may be interested in treatment effects for subpopulations, which can be calculated through CATE estimates. These subpopulations can be learned directly from the data or defined by several criteria, ranging from demographic strata or existing clinical heuristics with the goal of creating groups for which the treatment effect and goals are expected to be similar.

#### Direct and Indirect Stratification

CATE can be calculated *via* population stratification. The idea is to first stratify the population on *f*(*X*), i.e., a function of patient covariates X, into subpopulations. Then CATE for each subpopulation is calculated as the difference between the two expected potential outcomes within that subpopulation. As in Morgan and Winship (35), it is mathematically expressed as


$$\begin{array}{l} \tau_{\text{CATE}}=\text{E}\left(\text{Y}|\text{A}=1,\text{ f}\left(\text{X}\right)\right)-\text{E}\left(\text{Y}|\text{A}=0,\text{f}\left(\text{X}\right)\right) \end{array}$$

Function *f*(*X*) can take on different forms. In the basic form *f*(*X*) = *X*, the population is stratified directly on covariate *X* as described in Imbens and Rubin (36), which we call direct stratification. With this approach, the covariates within each stratum (subpopulation) are similar in values across different patients. Suited for scenarios where subpopulations are predefined, this approach provides simple and transparent interpretation of the subpopulation but may lead to data sparsity in some stratum or violation of the positivity assumption. Function *f*(*X*) can take on a more complex function form, which we call indirect stratification. If *f*(*X*) = π(*X*), the population is stratified on propensity scores (12, 29). This approach alleviates the data sparsity problem, but the interpretation of subpopulations is less intuitive.

#### Data Driven Determination of Subpopulations

A subpopulation can be viewed as a subspace in the multi-dimensional covariate space. A data driven approach to calculate CATE partitions the covariate space into subspaces in a way that the treatment effect heterogeneity across subspaces is maximized. The resulting subspaces (or subpopulations) reflect the heterogeneity of the underlying data. Some subspaces may be wider or narrower in certain dimensions than others depending on how quickly the treatment effect changes along these dimensions, which is a desired property.

Machine learning models, due to their flexibility, are well-suited for this approach. One of such estimators is proposed in Athey and Imbens (37) based on the classification and regression tree (CART) (38). While a CART model minimizes a predefined loss function in associative studies, it maximizes heterogeneous treatment effect across leaves when used in causal inference. Different sets of samples are used for constructing the tree and for estimating the treatment effect for each subpopulation. Because of this, the approach is called an honest estimation.

In contrast to the approach in Athey and Imbens (37) where only one decision tree is used, the approach proposed by Breiman (39) estimates treatment effects based on the random forest model consisting of multiple decision trees (40).

These machine learning-based models are non-parametric and thus robust to model misspecification. They can capture the heterogeneity structure in the underlying data and reduce the variance of effect estimates in a subpopulation. However, the complexity of such models makes the results less explainable compared to simpler ones, creating obstacles for the medical community to widely adopt these models in clinical applications.

### Estimate ITE for Individual Patients

Treatment effects can be different not only across subpopulations, but across different patients as well. Due to the existence of such heterogeneity at individual patient level, ITE estimates are important for personalized medicine and have been increasingly gaining attention in healthcare (41). In the strictest sense, the ITE estimate is conditioning on an individual's characteristics so can be regarded as CATE. However, in this work, we review ITE as a distinct algorithm category separated from CATE. This decision emphasizes the fact that ITE targets individual patients, while CATE targets subgroups of patients.

Intuitively, ITE can be calculated as the difference between the two potential outcomes for a patient. One of the potential outcomes is missing but can be estimated with an outcome regression model, where the potential outcome is the dependent variable and the covariates are the independent variables. In essence, such an outcome regression model fits a function to estimate the regression surface (or outcome surface) in the covariate space using observed patient outcome samples. Note that the function used in outcome regression can be linear, non-linear, or even non-parametric, depending on the underlying data structure. There are two approaches to fit the model, based on whether the samples from the treated and control group are pooled together in the training step.

#### One Regression Function

To estimate ITE, we can fit one regression function using pooled samples from both the treated and the control group and regard the treatment assignment *A* as one of the independent variables, as shown in the equation below,


(6) $$\begin{array}{r} E\left(Y|X,A\right)=m\left(X,A\right) \end{array}$$

where *m*(*X, A*) estimates the potential outcome conditioned on *X* and *A*. Then the ITE estimate for patient *i* is calculated as *m*(*X*_*i*_, 1)− m(*X*_*i*_, 0). One example of such a model is the Bayesian Additive Regression Trees (BART) introduced in Hill (42), Chipman et al. (43), and Chipman et al. (44), where the authors constructed a set of trees using ensemble learning, and imposed a prior regularization to constrain each tree to be a weak learner. Another example is proposed in Foster et al. (45), where the authors used a random forest to fit *m*(*X, A*) to estimate ITE. The approach proposed in Nie and Wager (46) fits a single outcome surface first to isolate the impact of the treatment on the outcome, then fits a regression model where the ITE is the only independent variable.

The models fitting one outcome surface are well-suited for scenarios where the treatment effect is small. The analysis in Wendling et al. (47) validates the performance of the BART model using synthetic data based on two major healthcare databases in the United States and concludes that the smaller the ITE is (i.e., the closer the outcome surfaces are between the two treatment groups), the better such models perform. These models perform poorly if there are complex interactions between the treatment assignment and covariates, which makes the outcome surface *f*(·) very different for the treated and control groups. Such model drawbacks are studied in detail in Alaa and Schaar (48) and Hahn et al. (49).

#### Two Regression Functions

Instead of fitting one regression function, one can fit two separate functions for the treated and control groups to calculate ITE. In this case, the treatment variable does not need to be included as one of the independent variables in the model because the outcome difference between the two groups is captured with different model parameters. The two regression functions can be expressed as


(7) $$\begin{array}{r} {E\left(Y^{1}|X\right)=\;m}_{1}\left(X\right) \end{array}$$

and


(8) $$\begin{array}{r} {E\left(Y^{0}|X\right)=\;m}_{0}\left(X\right) \end{array}$$

for the treated (*A* = 1) and control (*A* = 0) group, respectively. The ITE estimate for patient *i* is then calculated as *m*_1_(*X*_*i*_)− *m*_0_(*X*_*i*_). Different base learners can be used for *m*_0_(*X*) and *m*_1_(*X*), as proposed in Athey and Imbens (37), Lu et al. (50), Powers et al. (51), and Künzel et al. (52).

The approach fitting two outcome surfaces separately is suited for the scenarios where the outcome surface is very different for different treatment groups. The downside of this approach is that some common patterns between the two groups get lost during model fitting. A multitask-learning estimator introduced in Alaa and Schaar (48) and Alaa and Schaar (53) fits two outcome surfaces separately but attempts to recover common underlying patterns between the treated and control group through a joint optimization for the two groups.

#### Estimate Error Bound

Several theories proposed in the literature study the error of the ITE estimate. The authors in Shalit et al. (54) derived a theoretical upper bound for the error, which is a sum of the standard generalization-error in the representation space and the error resulted from the distance between the two treatment group covariate distributions induced by the representation. An extension of this work (named context-aware importance sampling re-weighing) is proposed in Hassanpour and Greiner (55) to theoretically address the selection bias in observational datasets, leading to a solution that weights the samples in such a way that the covariate distribution imbalance between the treated and control group is reduced. Related to the theoretical works above, practical solutions based on deep learning were proposed to incorporate in the loss function the dissimilarity of the learned representations for the treated and control groups so that the error induced by such dissimilarity can be reduced (56–58).

## Clinical Applications of Causal Inference

Although there are a large number of causal inference techniques in the literature as we reviewed above, these techniques are not applied equally to solve real-world clinical problems. In this section, we review the patterns of how the various causal inference approaches are used in published clinical studies.

### Reporting Methods

In searching for published application papers of causal inference models, we follow the applicable guidelines in accordance with the Preferred Reporting Items for Systematic Reviews and Meta-Analyses (PRISMA) (59). The modified PRISMA flow charts for each category of causal inference models are in the Supplementary Material. Note that although we follow the PRISMA guidelines whenever deemed applicable to make our search systematic, the review in this section is not a systematic review in the strictest sense, as our goal is not to answer a well-defined and narrowly focused clinical question, but to gain general understanding of the application landscape of causal inference.

### Results

Below we list the most relevant published clinical applications for each of the causal models we have identified. If the application list is too long (more than 15 publications), we just list below the top 15 most cited ones according to Google Scholar due to space limitations. The total number of applications identified with the inclusion and exclusion criteria is given in the Supplementary Material.

#### Applications of ATE Estimators for the Whole Population

Propensity score-based models have been applied to study the effect of interruption of sedation on the death of the patient in Requena et al. (60), the effect of corticosteroids on mortality for patients with influenza A (H1N1pdm09) in Delaney et al. (61), the cardiovascular, bleeding, and mortality risks in elderly Medicare patients treated with certain drugs in Graham et al. (62), the association of animal and plant protein intake with all-cause and cause-specific mortality in Song et al. (63), the effect of nasal cannula therapy failure on mortality in Kang et al. (64), the prevalence of sarcopenia in COPD and its impact on health in Jones et al. (65), the safety and efficacy of digoxin in Ziff et al. (66), clinical outcomes after transapical or transfemoral transcatheter aortic valve replacement in Blackstone et al. (67) and many other health related issues in Chang et al. (68), Bangalore et al. (69), Kost and Lindberg (70), Grool et al. (71), Snowden et al. (72), Han et al. (73), and Prati et al. (74).

Applications of outcome regression-based models in clinical studies have been rare. In fact, we did not find any applications of this approach that meet our search criteria.

Doubly robust estimators have been widely applied in real-world clinical studies to determine the effect of sepsis on late mortality in Prescott et al. (75), the effect of proton pump inhibitors use on risk of death in Xie et al. (76), cardiovascular risks of testosterone replacement therapy in men with androgen deficiency in Cheetham et al. (77), the effectiveness of influenza vaccines among elderly people in Izurieta et al. (78), whether antifungal de-escalation leads to adverse outcome in Bailly et al. (79), the association of the use of transthoracic echocardiography with 28-day mortality in Feng et al. (80), the effect of risk assessment on clinical outcomes in Chaffee et al. (81), comparison of children currently and previously diagnosed with autism in Blumberg et al. (82), whether there is a causal link between the Magnet status of a hospital and the central-line-associated bloodstream infections in Barnes et al. (83), as well as a range of health-related issues from association of aspirin with hepatocellular carcinoma and liver-related mortality to effect of angiotensin on hemoglobin levels in Breslau et al. (84), Simon et al. (85), Ajmal et al. (86), Millett et al. (87), Reed et al. (88), and Kawasaki et al. (89).

#### Application of CATE Estimators

CATE estimators using stratification have been widely applied in clinical studies, for example, to analyze the adverse outcomes of underuse of β-Blockers in elderly patients in Soumerai et al. (90), the rate of mortality in patients receiving drug-eluting stents and undergoing coronary-artery bypass grafting in Hannan et al. (91), the effect of Hydroxychloroquine and tocilizumab therapy on mortality in COVID-19 patients in Ip et al. (92), medical therapy on long-term outcome in patients with myocardial infarction (93), the impact of female sex on clinical outcomes for Atrial Fibrillation in Kuck et al. (94), and a range of other clinical issues (95–104).

There are very few applications of the data driven approach in clinical studies. The recursive partitioning approach (37) is used to study the effect of fluoxetine in patients with a recent stroke in Graham et al. (105), the effect modification in a study of surgical mortality in Lee et al. (106).

#### Application of ITE Estimators

The applications of ITE estimators are very rare in the literature. The BART model is used to predict the papillary thyroid carcinoma in Guo et al. (107) and to study the consequences of contact with the criminal justice system for health in Esposito et al. (108).

### Methods

#### Search Strategy

Here we describe the search strategy we use to find the published clinical applications of a causal approach. First, we identify the paper in which the model is proposed. If multiple models hence multiple papers exist—there might be model variations, extensions, or improvements—we pick a paper that generated the most citations in Google scholar. We then search in Google Scholar for all the publications citing the identified paper, which we call the anchoring paper, and apply the inclusion and exclusion criteria described below to determine what papers should be included in the application list of the causal approach.

Note that this search strategy is not exhaustive and is not intended to be a scoping review. Using the anchoring paper, we can only identify a subset of the application papers in a causal inference category. Our goal is not to precisely count the number of all applications, but to understand the extent to which different causal models are applied clinically. Accordingly, our strategy is to sample a limited number of publications, but in a systematic way, so that our search is manageable but still reflective of the application landscape in this field.

#### Inclusion and Exclusion Criteria

For each category of the causal inference approach, we search for publications that cite the anchoring paper in Google Scholar. In the returned result, we exclude any records not in the healthcare domain, which are those that do not contain any of these keywords: medicine, hospital, patient, clinics, healthcare, physician, and disease. We then screen the titles and abstracts of the remaining papers and exclude those not pertaining to applications. Most of the papers eliminated in this step are about models and algorithms related to the causal inference model described in the anchoring paper. The papers remaining after this step are clinical applications that cite the anchoring paper. However, the anchoring paper can be cited in many ways: it can be mentioned in the related work section; it can be cited in the discussion section; or it can be used to derive findings and insights. We proceed to read the papers that are cited more than 10 times, focusing on the section where the anchoring paper is cited. We include the paper in the final application list if the model in the anchoring paper is used as the method (or one of the methods) to draw conclusions, derive findings, or gain insights.

### Observations

A pattern emerged from surveying and analyzing the applications of causal models in healthcare: although state-of-the-art machine learning-based approaches have been consistently used to improve causal inference techniques algorithmically and generated excitement in the medical research community, these approaches have not been widely adopted in clinical studies. In contrast, simpler approaches based on propensity scores have been widely applied to solve real-world clinical problems. This conclusion is evident from the citation numbers in the Supplementary Material: while the number of machine learning applications, such as those based on models in Rubin (30) and Athey and Imbens (37), is in single digit at most, the number of applications based on propensity scores (12) is in hundreds.

We suggest several potential explanations for the wider adoption of propensity score-based approaches. First, the gold standard for causal inference in healthcare has long been the Randomized Controlled Trial (RCT). Propensity score-based approaches provide methods that mimic RCTs while using large-scale, observational data. Secondly, as we mapped out in Table 1, propensity score-based approaches offer relatively low variance at the risk of higher bias, which is consistent with medical applications where the goal to minimize patient harm outweighs the potential to increase benefits for a few. Third, there is an issue of timing, newer methods have simply been in existence for a shorter period of time and therefore have had less chance for adoption. However, this answer is least satisfying because many of the newer machine learning approaches have been successfully applied in many other fields such as gaming, online shopping, and advertising (4). Additionally, many machine learning-based causal models have been around for a long time. For example, as of the time this paper is written, the BART model (44) has existed for over a decade, and yet we have not seen many clinical applications of it. A fourth potential reason for lower adoption of purely machine learning based approaches is method explainability. In healthcare, where lives are frequently at stake, the requirement for methods that are explainable to a wide audience are significantly higher than other fields, where effectiveness alone may be sufficient.

**Table 1:** Summary of causal inference approaches in healthcare.

**Target-Population intervention sizes**	**Estimator types**	**Models and algorithms**	**Advantages**	**Disadvantages**	**Variance**	**Bias**	**Clinical application patterns and references**
		Propensity scores-based, propensity score matching and IPTW	Simple, transparent, mimic clinical trials	Model can be misspecified			Widely used (60, 68)
Whole population	ATE	Outcome regression, variations of G-computation	No need to estimate propensity score	Model can be misspecified	Low	High	Few applications
		Doubly robust estimator, targeted maximum likelihood estimator	Efficient, doubly robust property	Yield biased estimate if both models are misspecified			Widely used (75, 84)
		Direct stratification	Easy to interpret	Data sparsity proble			Widely used (90, 95)
Sub population	CATE	Indirect stratification, propensity score-based approach	Robust, easy to satisfy positivity assumption	Subpopulation hard to interpret	Medium	Medium	
		Data driven, tree based algorithms	Low variance within subpopulation	Subpopulation hard to interpret	Medium	Medium	Few applications (105, 106)
		Fit one outcome surface, BART model etc	Capture common underlying data structure	Not flexible, especially when the outcome surfaces are very			Few applications (107, 108)
Individuals	ITE			different in distinct groups	High	Low	
		Fit two outcome surfaces	Flexible, allow for different data structure in groups	Does not capture common data pattern in two groups			

We believe that lower historical adoption of more modern observational causal inference approaches is sensible, but that it also represents a gap in the field, especially given the potential promise of more personalized medicine using ITE-type estimators. This gap could potentially be closed in the near future by collaborative pairing of biostatisticians and machine learning scientists with clinicians.

## Flowchart for Algorithm Selection

**Figure 1:**
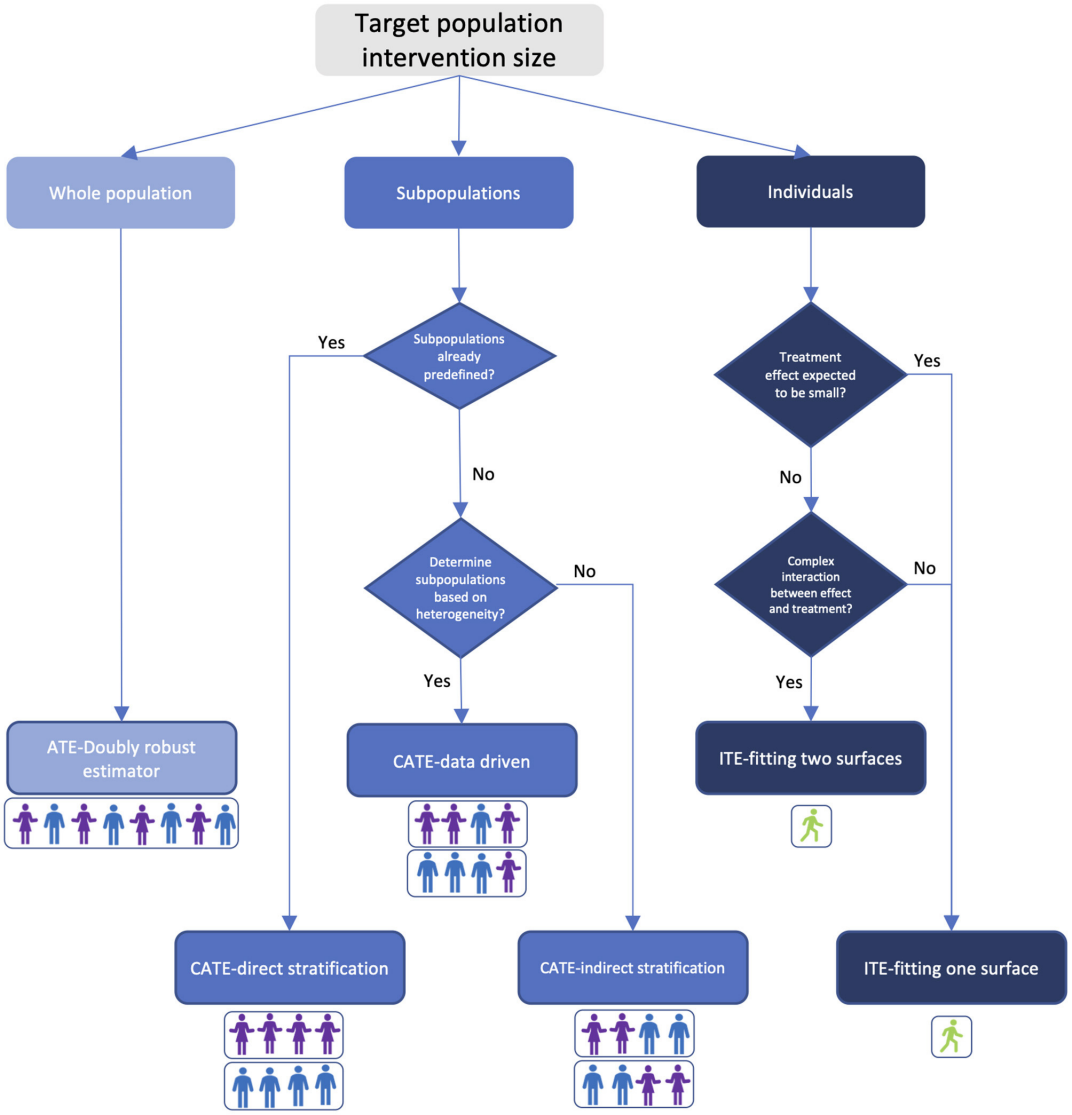
Treatment effect estimator selection guide based on target-population intervention size and prior knowledge. Colors in the figure indicate bias-variance tradeoff. Light blue: high bias and low variance; blue: medium bias and variance; dark blue: low bias and high variance. Person icons under each estimator illustrate the composition of the targeted population.

In this section we provide a guide in Figure 1 to help the healthcare community choose which algorithm to use in estimating treatment effects based on the target-population intervention sizes, domain knowledge about the treatment, and track record of healthcare applications of the algorithm. While every problem is unique, and individual judgement must always be exercised, this flowchart can act as a starting point to determine which algorithmic approach may be most appropriate.

## Discussion

In this paper we reviewed the literature on causal inference with a focus on clinical settings, in light of recent advances in machine learning and large scale EHR adoption. With this review, the algorithm selection guide, and the summary table, we hope to help researchers and healthcare stakeholders gain better understanding of causal inference and make informed decisions on what estimator to use in their daily practices when many choices are on the table.

We have observed that sophisticated causal models based on state-of-the-art machine learning have not been widely applied in clinical studies for a myriad of reasons such as lack of similarity to RCTs and explainability (Section Clinical Applications of Causal Inference), computational intractability of these models, and the healthcare participants being highly conservative when adopting new models. To address the same issue and improve model transparency, a MI-CLAIM check list in Norgeot et al. (109) was proposed regarding the study design of projects, preparation and usage of data, model selection, performance evaluation, model validation, and data pipelines. Our review stresses the importance to follow these guidelines to promote trust on sophisticated models among clinical practitioners.

There are some limitations of the review. First, it may not be exhaustive and include every approach. Causal inference is a very broad topic. While we can limit our review to a specific topic to be exhaustive, it is also important to survey the entire field of causal inference, thus sacrificing the completeness to some degree. Second, causal inference approaches are grouped into ATE, CATE, and ITE categories in this review. These categories might not be mutually exclusive. Such classification, however, does provide an intuitive way for medical professionals to understand causal inference from patient perspectives. Third, there are certain limitations of using citations to rank the applications. For instance, an algorithm applied in clinics might not have been published. Additionally, for a recent work, the citation number might be low, and might not accurately reflect the application potential of the work. Fourth, Table 1 and Figure 1 do not cover all the details of choosing an algorithm, nor do they lead a user to a specific algorithm. They were designed to provide all healthcare participants with an initial but intuitive guide on what family of algorithms to choose for their studies. Finally, our search to find published applications of causal models may not be exhaustive. The search results show that the application disparity of different models is so huge that a different (and potentially more comprehensive) search strategy will unlikely change our conclusions and insights in any significant way.

There is a view in the literature that causal inference is just plain statistical inference, especially after the causal assumptions and parameters are identified (110). The role of causal inference with respect to statistical analysis remains a debate. This debate is out of scope for this paper. We refer to the reviewed models as causal inference models without endorsing any particular view on this matter, but simply use this name to refer to the statistical inference models that reveal causal relationships.

In summary, we reviewed a diverse and complex field of causal inference applied in health care. We distilled the many approaches into three algorithmic families based on the target-population intervention size. We explained the approach type, population size, and bias-variance tradeoff. We then investigated the clinical application of each of the approaches. We finally consolidate all the information into an algorithm selection guide for both researchers and other healthcare stakeholders to decide on which algorithm is applicable to their studies.

## Author Contributions

JS conducted the research and developed the figures. BN conceived of the research topic and wrote the manuscript. Both authors contributed to the article and approved the submitted version.

## Funding

The authors JS and BN are employed by Anthem, Inc. The funder had no other involvement in the study design, collection, analysis, interpretation of data, the writing of this article, or the decision to submit it for publication.

## Conflict of Interest

JS and BN are employed by Anthem, Inc.

## Publisher's Note

All claims expressed in this article are solely those of the authors and do not necessarily represent those of their affiliated organizations, or those of the publisher, the editors and the reviewers. Any product that may be evaluated in this article, or claim that may be made by its manufacturer, is not guaranteed or endorsed by the publisher.
