# Supplementary material for: Learning Causal Effects From Observational Data in Healthcare: A Review and Summary
Source: Front Med (Lausanne). 2022 Jul 7;9:864882. doi: 10.3389/fmed.2022.864882 (PMC9300826; doi:10.3389/fmed.2022.864882)
Supplement: Supplementary file 1 [file Data_Sheet_1.docx]

Supplementary Material

# Supplementary anchoring papers and PRISMA flow charts.

Here we show the anchoring papers and PRISMA flow charts for identifying applications of each causal inference category.

*Application of ATE estimators*

29568 records identified through google scholar search

13868 records not in healthcare excluded through keywords search

15700 records screened as titles and abstracts

12560 records included for eligibility

12189 records excluded due to approach not being used in statistical analysis or citation < 10 times

371 records included in application list

3140 records not applications excluded

**Supplementary Chart 1.** Chart for ATE propensity score-based models. Anchoring paper: (1)

1053 records identified through google scholar search

783 records not in healthcare excluded through keywords search

270 records screened as titles and abstracts

148 records included for eligibility

148 records excluded due to approach not being used in statistical analysis or citation < 10 times

122 records not applications excluded

0 records included in application list

**Supplementary Chart 2.** Chart for ATE outcome regression-based models. Anchoring paper: (2)

481 records identified through google scholar search

247 records not in healthcare excluded through keywords search

234 records screened as titles and abstracts

156 records included for eligibility

90 records excluded due to approach not being used in statistical analysis or citation < 10 times

47 records included in application list

78 records not applications excluded

**Supplementary Chart 3.** Chart for ATE doubly robust estimators. Anchoring paper: (3)

*Application of CATE estimators*

4507 records identified through google scholar search

2607 records not in healthcare excluded through keywords search

1900 records screened as titles and abstracts

1463 records included for eligibility

1357 records excluded due to approach not being used in statistical analysis or citation < 10 times

106 records included in application list

437 records not applications excluded

**Supplementary Chart 4.** Chart for CATE estimators using stratification. Anchoring paper: (4)

837 records identified through google scholar search

645 records not in healthcare excluded through keywords search

192 records screened as titles and abstracts

34 records included for eligibility

32 records excluded due to approach not being used in statistical analysis or citation < 10 times

2 records included in application list

158 records not applications excluded

**Supplementary Chart 5.** Chart for CATE using data driven approach. Anchoring paper: (5)

*Application of ITE estimators*

1282 records identified through google scholar search

962 records not in healthcare excluded through keywords search

320 records screened as titles and abstracts

146 records included for eligibility

144 records excluded due to approach not being used in statistical analysis or citation < 10 times

2 records included in application list

174 records not applications excluded

**Supplementary Chart 6.** Chart for ITE estimators. Anchoring paper: (6)

**References** x

| 1. | Rosenbaum PR, Rubin DB. The central role of the propensity score in observational studies for causal effects. Biometrilca. 1983; 70(1): p. 41-55. |
| --- | --- |
| 2. | Rubin DB. Using Multivariate Matched Sampling and Regression Adjustment to Control Bias in Observational Studies. Journal of the American Statistical Association. 1979; 74(366a): p. 318-328. |
| 3. | Funk MJ, Westreich D, Wiesen C, Stürmer T, Brookhart MA, Davidian M. Doubly robust estimation of causal effects. Am J Epidemiol. Apr 2011; 173(7): p. 761-7. |
| 4. | Rosenbaum PR, Rubin DB. Reducing Bias in Observational Studies Using Subclassification on the Propensity Score. Journal of the American Statistical Association. 1984; 79(387): p. 516-524. |
| 5. | Athey S, Imbens G. Recursive partitioning for heterogeneous causal effects. Proceedings of the National Academy of Sciences. 2016; 113(27): p. 7353-7360. |
| 6. | Chipman HA, George EI, McCulloch RE. BART: Bayesian Additive Regression Trees. The Annals of Applied Statistics. 2010; 4(1): p. 266-298. |

x
